# Objective characterization of hip pain levels during walking by combining quantitative electroencephalography with machine learning

**DOI:** 10.1038/s41598-021-82696-1

**Published:** 2021-02-04

**Authors:** Atsushi Kimura, Yasue Mitsukura, Akihito Oya, Morio Matsumoto, Masaya Nakamura, Arihiko Kanaji, Takeshi Miyamoto

**Affiliations:** 1 Department of Orthopedic Surgery, Keio University School of Medicine, 35 Shinano-machi, Shinjuku-ku, Tokyo, 160-8582 Japan; 2 Department of Advanced Therapy for Musculoskeletal Disorders II, Keio University School of Medicine, 35 Shinano-machi, Shinjuku-ku, Tokyo, 160-8582 Japan; 3 Department of Musculoskeletal Reconstruction and Regeneration Surgery, Keio University School of Medicine, 35 Shinano-machi, Shinjuku-ku, Tokyo, 160-8582 Japan; 4 Department of Technology and Engineering, Keio University, Yokohama, 2238532 Japan; 5 Department of Orthopedic Surgery, Kumamoto University, 1-1-1 Honjo, Chuo-ku, Kumamoto, 860-8556 Japan

**Keywords:** Diseases, Biological techniques

## Abstract

Pain is an undesirable sensory experience that can induce depression and limit individuals’ activities of daily living, in turn negatively impacting the labor force. Affected people frequently feel pain during activity; however, pain is subjective and difficult to judge objectively, particularly during activity. Here, we developed a system to objectively judge pain levels in walking subjects by recording their quantitative electroencephalography (qEEG) and analyzing data by machine learning. To do so, we enrolled 23 patients who had undergone total hip replacement for pain, and recorded their qEEG during a five-minute walk via a wearable device with a single electrode placed over the Fp1 region, based on the 10–20 Electrode Placement System, before and three months after surgery. We also assessed subject hip pain using a numerical rating scale. Brain wave amplitude differed significantly among subjects with different levels of hip pain at frequencies ranging from 1 to 35 Hz. qEEG data were also analyzed by a support vector machine using the Radial Basis Functional Kernel, a function used in machine learning. That approach showed that an individual’s hip pain during walking can be recognized and subdivided into pain quartiles with 79.6% recognition Accuracy. Overall, we have devised an objective and non-invasive tool to monitor an individual’s pain during walking.

## Introduction

Pain was defined as an unpleasant sensory and emotional experience associated with actual or potential tissue damage, or described in terms of such damage by the International Association for the Study of Pain in 1981^1^. Various types of pain have been described^1–3^; for example, nociceptive pain is due to stimulation of nociceptors by pain-producing substances such as bradykinin and prostaglandin or by ATP produced by inflammation. By contrast, neuropathic pain is caused by nerve injury or disfunction^2^. Nonetheless, pain causes distress, mood changes, or even depression. Pain is also detrimental to workforce productivity^4,5^, and it is reported that workers experiencing pain lost an average of 4.6 work hours per week in the USA^6^. Back pain in workers 40–65 years of age reportedly cost employers an estimated 7.4 billion US dollars per year^7^. Thus, managing pain is mandatory for both individuals and society.

Managing pain requires objective diagnosis, estimation and evaluation of treatment effectiveness. However, it is difficult to evaluate pain quality and intensity objectively. To date, various tools have been developed to characterize pain, namely the visual analog scale (VAS), numerical rating scale (NRS), face rating scale (FRS) and McGill questionnaire^8–10^, but it remains difficult to evaluate pain with objectivity. For the NRS, patients are asked to rate pain using an eleven-point scale ranging from 0 (no pain) to 10 (severest pain) along a horizontal line. The NRS is one of the most commonly used pain scales in medicine^11,12^ and has been used to evaluate hip pain in several studies^13–15^. Devices and software have also been developed to monitor pain using weak, painless current or functional magnetic resonance imaging (MRI)^16–21^. These approaches are useful to monitor pain at rest but cannot be applied to evaluate pain during motion, such as walking^21,22^. Electroencephalogram (EEG) recording has also been applied to characterize chronic pain, but since EEG recordings require use of multiple electrodes (> 4), monitoring using EEG has been limited to periods of rest, sensory stimulation or performance of cognitive tasks^23^.

Brain waves are rhythmic or repetitive patterns of electronic signals recorded by electrodes, which reflect neural activity in the central nervous system^24–27^. Brain wave frequency and amplitude change and are influenced by numerous factors^24,25,27^. Some diseases can be diagnosed by particular brain wave or EEG patterns^26,27^, and pain also can alter an EEG pattern^23^. EEG pattern is also affected by electrode position, and thus, specific methods have been devised to position electrodes, such as the 10–20 system^28^. The prefrontal region, defined as the Fp1 position by that system, is reportedly responsive to pain^29^. Although this approach can be influenced by motion artifacts, monitoring brain waves is a non-invasive method, can be continuously recorded, and is a useful tool if artifacts are minimized.

Arthritis is a major cause of loss of productive work time^30^, accounting for a reported loss of 7.11 billion US dollars per year in the US^30^. Hip arthritis develops by loss of articular cartilage in hip joints owing to excess weight, acetabular dysplasia, trauma or rheumatoid arthritis^31–33^. Hip osteoarthritis (OA) is classified by Kellgren-Lawrence (KL) grade based on radiographic estimation of the joint space narrowing^34,35^. KL grade 0 is normal, while grade 4 is most severe with joint space narrowing over 75% of the area. High grade cases with severe pain and difficulties in activities of daily living (ADL) are often treated with total hip arthroplasty (THA), which can also relieve pain in patients with osteonecrosis of the femoral head^36^. Such surgical treatment for hip arthritis not only eases pain but improves patients’ quality of life (QOL)^32,33,37,38^.

Here, we devised a means to objectively assess hip pain during walking by monitoring brain waves in subjects wearing a brain wave sensor with a single electrode placed at the Fp1 position. We then subjected patients to brain wave analysis during a five-minute walk before and after THA surgery. We also monitored hip pain by NRS, and analyzed the relationship between those values and changes in brain wave patterns. We show that brain wave amplitude differed significantly between patients with hip pain and those without, and that those differences enabled us to objectively determine the degree of pain using machine learning. Our system represents a useful, non-invasive tool to estimate hip pain during walking.

## Materials and methods

### Subjects

Subjects were hip OA or osteonecrosis patients who underwent initial THA between December 2017 and June 2018 at our hospital. Forty subjects were invited to participate, and written informed consent was obtained from all. Seventeen were excluded from the study based on the following criteria: (1) refusal to undergo a follow-up examination, (2) incomplete datasets, or (3) non-standard course with perioperative complications. Twenty-two subjects were Japanese men and women, and one was an Italian woman. This study was approved by an ethics committee at Keio University School of Medicine and was carried out in accordance with clinical study guidelines.

### Measurements

Age at the time of surgery, sex, height, body weight, body mass index (BMI) calculated from body weight and height data, OA severity, and condition of the contralateral hip were assessed in all subjects. In this study, patients rated pain in the site targeted by surgery using the NRS scale and also underwent EEG recording using a wearable device on admission and three months after operation. The surgical site analyzed in subjects who underwent bilateral THA was defined as the more painful hip.

### Quantitative EEG analysis

**Figure 1:**
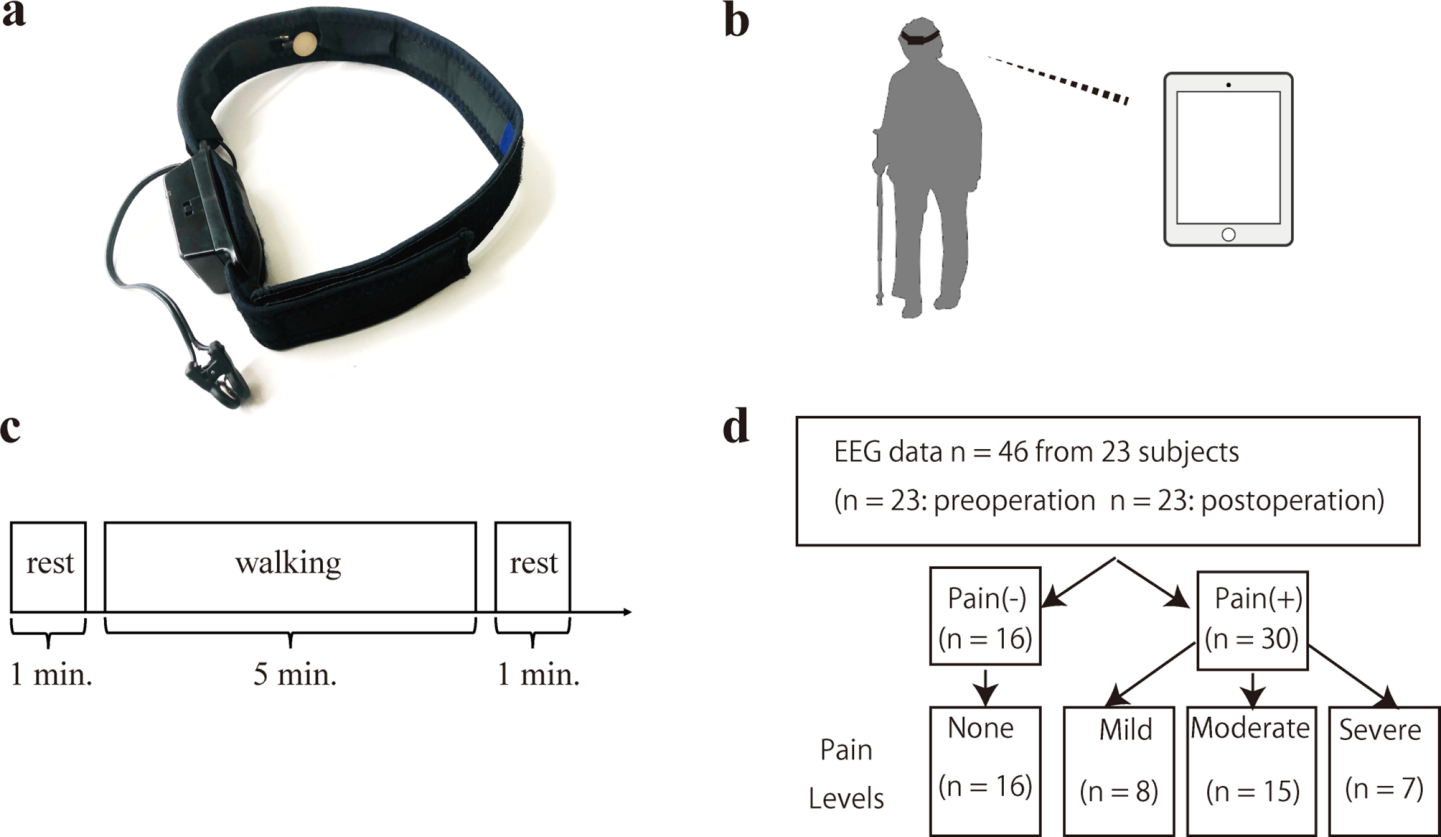
Development of an EEG recording system applicable to walking. (**a**) The wearable EEG recording device, MindWave Mobile II (Neurosky), used in this study to record the EEG from electrode position Fp1. Conductive material reference and ground electrodes were contained within the ear clip. (**b**)Wireless control of the device with an iPad. Subjects were able to walk with hands free. (**c**) Task diagram. The task consisted of a one-minute rest block with subjects in a sitting position with eyes closed, then a walking block of 5 min, and finally a rest block like the first. For the walking block, we encouraged patients to walk at a uniform pace and in a way that simulated their daily habits. (**d**) Pain classification flowchart. We asked about hip pain while recording EEG, before and after THA surgery. We collected 46 sets of NRS data from 23 subjects, and sets were subdivided into Pain (–) (NRS 0, *n* = 16) or Pain ( +) (NRS ≥ 1, *n* = 30) groups. Pain ( +) groups were subdivided into quartiles, as indicated. Each EEG data recording session of 300 s from 46 individuals was divided into 10 data sets of 30 s. Thus the total number of datasets analyzed was 460.

EEG was recorded in subjects equipped with a wearable device (the MindWave Mobile II (Neurosky)), before and three months after THA surgery (Fig. 1). A single electrode was placed over the Fp1 region based on the 10–20 International System, and ground and reference potentials were connected by an ear clip (Fig. 1a). Recorded EEG was transferred to an iPad wirelessly via Bluetooth (Fig. 1b). An EEG at 0–40 Hz was recorded during a 7-min walk, and data from a 5-min (300 s) interval was analyzed by excluding the first and last minutes (Fig. 1c). Recorded raw data in the 5-min interval was normalized as average 0 and standard deviation 1 to eliminate individual differences and compare amplitude in each brain wave frequency among subjects. A bandpass filter was adopted as 0.5 to 36 Hz, and an electromyogram was eliminated, as described^23,25^. Remaining data were subdivided into EEG of every 30 s, and fast Fourier transformation was applied to calculate the average power spectrum. The resulting average power spectrum in each frequency ranging from 1 to 35 Hz was determined as each feature of EEG.

### Prediction of hip pain in individuals based on EEG features

All 46 sets of NRS data from 23 subjects (before and three months after THA) were subdivided into two groups, Pain (−) (NRS = 0) and Pain ( +) (NRS ≥ 1) (Fig. 1d), and the power spectrum in each brain wave frequency was compared between groups and analyzed statistically using Welch’s t-test. Then, pain levels based on NRS were subdivided into quartiles: none (NRS 0), mild (NRS 1–3), moderate (NRS 4–6) and severe (NRS 7–10), and the power spectrum in each frequency compared among levels. Statistical analysis was performed using Welch’s t-test to compare pain ( +) and pain (−) groups, and by Dunn’s test to compare the 4 pain grade groups. That comparison was made using analysis of variance based on the Kruskal–Wallis test. Resultant *p* values were corrected by Bonferroni correction to evaluate significance. A Support Vector Machine (SVM) using a Radial Basis Functional Kernel (RBF kernel), a function used in machine learning, was applied to make a decision using the identification that maximizes the margin indicating the distance of the data (support vector) located at the minimum distance from the identification surface. Precision or Recall was determined by dividing the number of identical sets of predicted (based on machine learning) and actual (based on NRS) pain levels by a whole number of sets in each predicted or actual pain level, respectively. Accuracy was calculated using a confusion matrix and determined by dividing identical sets of predicted and actual pain levels in both dimensions by whole sets.

## Results

### Basic characteristics of the subjects

**Figure 2:**
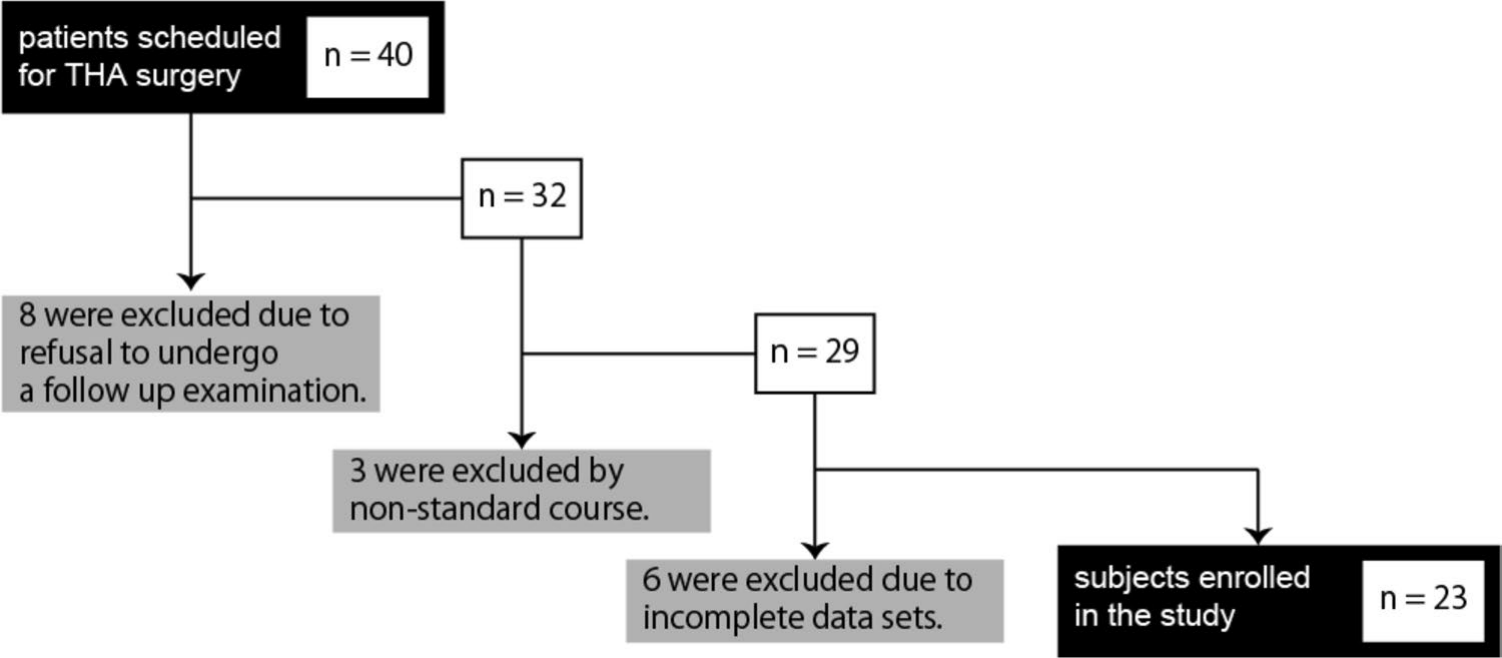
Flow chart of study subjects. Eight patients were excluded due to refusal to undergo follow-up examination, three were excluded by non-standard course such as re-operation, and six were excluded due to incomplete data sets. Most patients who refused undergo an examination were unable to walk continuously for 5 min.

We invited 40 patients who had been admitted to our hospital suffering from hip pain due to OA or osteonecrosis and were scheduled for THA surgery. Eight were excluded due to refusal to undergo follow-up examination, 3 were excluded based on non-standard course such as re-operation, and 6 were excluded owing to incomplete data sets, leaving 23 enrolled in the study (Fig. 2): 18 female and 5 male, aged 44–80 years old (64.6 ± 11.9) (Table 1). Characteristics of the original 40 patients were similar to those of remaining 23 (Supplementary Table1). In general, OA and osteonecrosis were more common in females than males^38–40^, and thus females underwent THA surgery more frequently. Indeed, the percentage of females among patients who underwent THA surgery at our hospital was 73.8%, 77.2% and 75.7% in 2017, 2018 and 2019, respectively. Subject body mass index ranged from 15.4 to 28.8 kg/cm^2^ (22.2 ± 3.9) (Table 1). The Kellgren-Lawrence (KL) classification, which characterizes radiographic OA grade, was assessed: 2 patients were grade 2 (8.7%), 7 grade 3 (30.4%) and 14 were the severest grade 4 (60.9%) (Table 1). One osteonecrosis patient was enrolled in this study, and the KL classification was 2. Pain in that patient was considered to emerge from osteonecrosis rather than due to changes in radiographic OA. Ten (43.4%) hips were defined as OA with developmental dysplasia of the hip based on a Sharp’s angle of > 45°^41^. The contralateral side of the hip joint in each subject was assessed as normal (8 joints), OA (10 joints), or post-THA surgery (5 joints). Seven of the 10 contralateral OA joints that underwent simultaneous THA were not evaluated for this study as they were judged the less painful of the two.

**Table 1:** Characteristics of patients. Continuous values are expressed as mean ± standard deviation (range).

Parameters	N = 23
Age at THA (y)	64.6 ± 11.9 (44–80)
Female/Male, n (%)	18(78.3)/5(21.7)
Height (cm)	156 ± 7.51(145–172)
Body weight (kg)	54.3 ± 9.97(36.7–73.2)
BMI (kg/m^2^)	22.2 ± 3.9 (15.4–28.8)
Severity of OA (K/L grade).1/2/3/4, n (%)	0/2(8.7)/7(30.4)/14(60.9)
OA due to DDH, n (%)	10(43.4)
Contralateral hip, healthy/OA/after THA, n (%)	8(34.8)/10(43.5)/5(21.7)

THA, total hip arthroplasty; BMI, body mass index; OA, osteoarthritis; K/L, kellgren-Lawrence; DDH, developmental dysplasia of the hip.

### Brain wave amplitude differs significantly between subjects with or without hip pain

**Figure 3:**
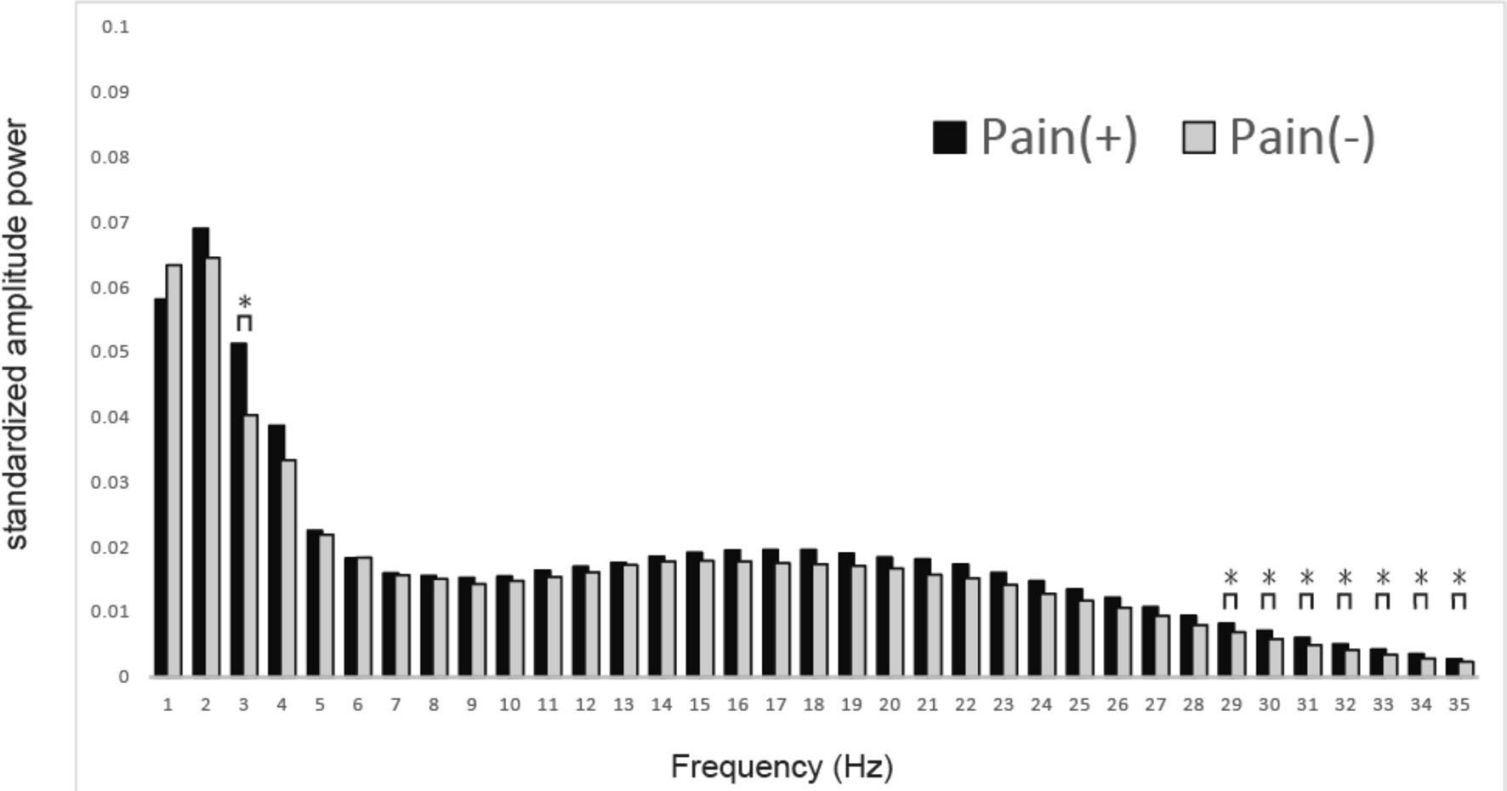
Brain wave amplitude at various frequencies differs significantly between Pain (−) and (+). Brain wave amplitude was recorded during a five-minute walk in patients with (Pain (+), NRS > 1, *n* = 30) or without (Pain (−), NRS 0, *n* = 16) hip pain, and the difference in amplitude at frequencies ranging from 1 to 35 Hz was compared between groups. Brain wave amplitude was significantly higher in some frequencies in the Pain (+) than Pain (−) groups (*p < 0.05).

First, we asked subjects to rate hip pain by NRS before and three months after THA surgery. To do so, we collected 46 sets of NRS data from 23 subjects (before and after surgery). Subject NRS scores before surgery ranged from 1 to 10 (mean 5.70 ± 2.36), and those numbers improved significantly after surgery, ranging from 0 to 4 (mean 0.74 ± 1.32) (*p* < 0.001). We also recorded EEG in the 23 participants before and three months after surgery by recording from a single electrode placed over the prefrontal Fp1 region, since that region reportedly functions in sensing pain^29^ (Fig. 1a). The recorded EEG was transferred wirelessly to an iPad, allowing subjects to walk normally with their free hands (Fig. 1b) during the five-minute period of EEG recording (Fig. 1c; see Methods). Forty-six sets of NRS data were subdivided into Pain (–) (NRS 0, *n* = 16) or Pain (+) groups (NRS ≥ 1, *n* = 30), and the amplitude of each frequency ranging from 1 to 40 Hz was compared between groups (Fig. 1d). We observed that amplitude was significantly higher in some frequencies in the Pain (+) than Pain (−) group (Fig. 3).

### Pain levels can be recognized by an EEG pattern

**Figure 4:**
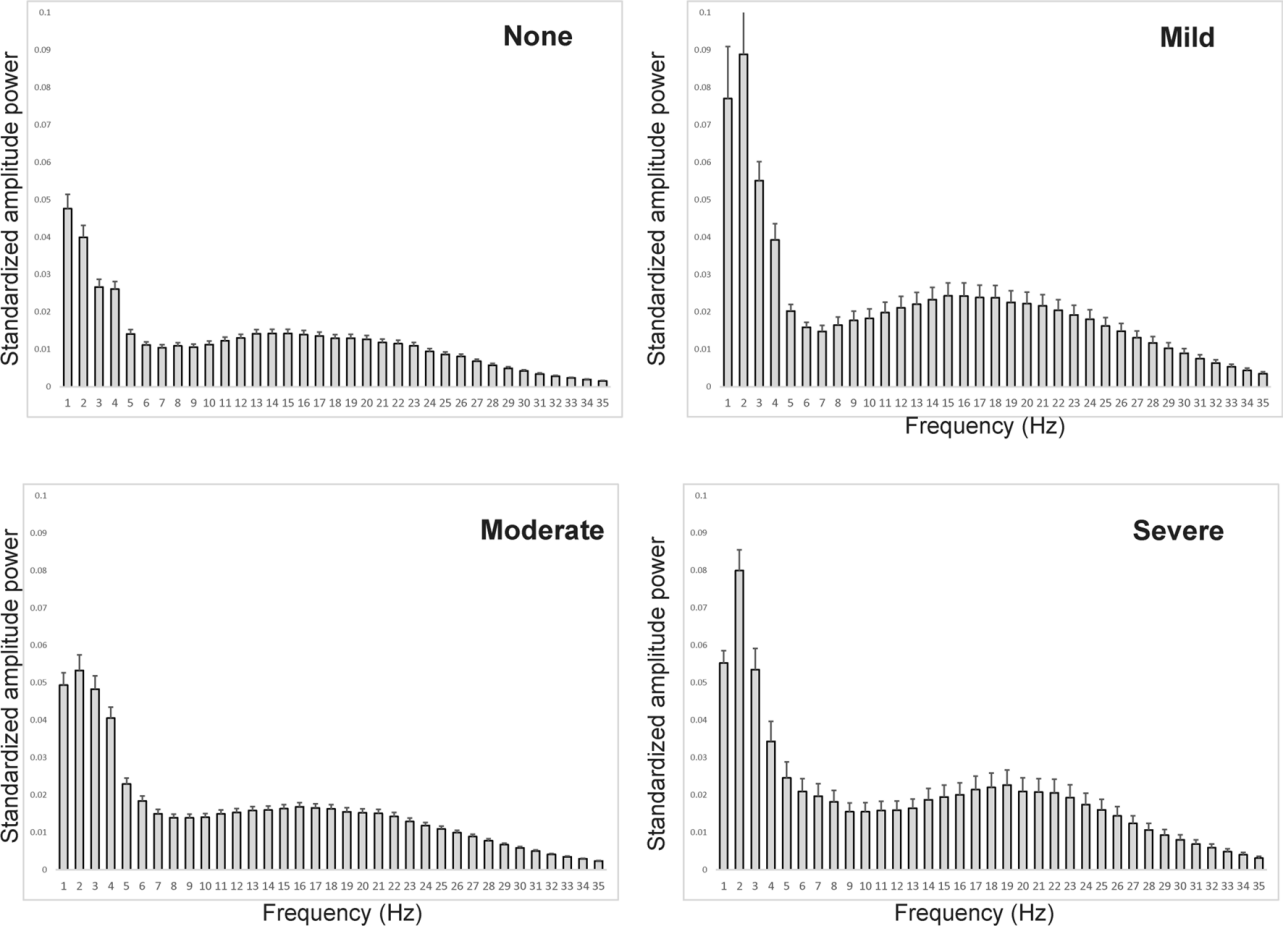
Brain wave amplitude differs significantly among pain levels. Brain wave amplitude was recorded during a five-minute walk in patients with or without hip pain. Each pain level was subdivided into the following quartiles based on the NRS: none (NRS 0, *n* = 16), mild (NRS 1–3, *n* = 8), moderate (NRS 4–6, *n* = 15) and severe (NRS 7–10, *n* = 7), and the power spectrum at frequencies ranging from 1 to 35 Hz was analyzed.

As pain had a significant effect on brain wave amplitude (Fig. 3), we divided pain levels into quartiles based on the NRS score: none (NRS 0, *n* = 16), mild (NRS 1–3, *n* = 8), moderate (NRS 4–6, *n* = 15) or severe (NRS 7–10, *n* = 7). We then compared brain wave amplitude at each frequency among quartile pain levels (Fig. 4 and Table 2). From 1–35 Hz, amplitude differed significantly between pain levels, as shown in Fig. 4 and Table 2.

**Table 2:** Differences in brain wave amplitude of several frequency bands between pain levels.

Pain levels to compare	Frequency(Hz)	*P* value
None vs Moderate	1 Hz	0.003
	2 Hz	0.002
None vs Severe	5 Hz	0.005
	6 Hz	0.008
	11 Hz	0.036
	12 Hz	0.016
	13 Hz	0.008
	14 Hz	0.007
	15 Hz	0.011
	16 Hz	0.031
	18 Hz	0.034
	24 Hz	0.050
Mild vs Moderate	1 Hz	0.001
	2 Hz	< 0.001
Mild vs Severe	12 Hz	0.030
	14 Hz	0.036
	15 Hz	0.039
Moderate vs Severe	2 Hz	< 0.001
	4 Hz	0.010
	11 Hz	0.041
	12 Hz	0.026
	13 Hz	0.035

Amplitude power in each frequency was compared among quartile pain levels.

The amplitude power of 10 frequency bands was significantly different between pain levels “None” and “Severe”. Similarly, 2 bands, 3 bands, 2 bands and 5 bands were significantly different between pain levels “None” and “Moderate”, “Mild” and “Severe”, “Mild” and “Moderate” and “Moderate” and “Severe”, respectively. There were no significant between “None” and “Mild”.

Statistical analysis was undertaken using Student’s t-test and the resultant *p* values were corrected by Bonferroni correction to evaluate the significance.

These results suggest that pain levels can be recognized objectively by an EEG pattern. Thus, we asked whether pain levels could be recognized by the process of feature extraction of EEG by a Support Vector Machine (SVM) using Radial Basis Functional (RBF) Kernel, a function used in machine learning. As a result, Precision in predicting “none”, indicating that matching of predicted pain levels of “none” based on machine learning with actual pain of “none” as measured by NRS, was 83.95% (Tables 3 and 4). Similarly, Precision in predicting “mild”, “moderate” and “severe” pain was 69.74, 79.75 and 79.69%, respectively. Moreover, Recall relevant to “severe” pain, indicating matching of actual pain based on NRS with predicted “severe” pain based on machine learning, was 72.89% (Tables 3 and 4). Similarly, Recall in “none”, “mild” and “moderate” conditions was 85.00, 66.25 and 84.00%, respectively (Tables 3 and 4).

**Table 3:** Results of pain levels classification using the SVM classifier.

		Output results			
		None	Mild	Moderate	Severe
Ground truth label	None	136	9	11	4
	Mild	9	53	12	6
	Moderate	11	10	126	3
	Severe	6	4	9	51

Each results were determined by dividing the number of identical sets of predicted (by the machine learning) and actual (NRS) pain levels by whole number of sets in each predicted or actual pain level, respectively.

**Table 4:** Values of the generalization performance of the classification using the SVM classifier.

	Precision	Recall	F1-score	Overall CA
None	0.8395	0.8500	0.8477	0.796
Mild	0.6974	0.6625	0.6795	
Moderate	0.7975	0.8400	0.8182	
Severe	0.7969	0.7286	0.7612	

CA: classification accuracy.

Finally, we analyzed Accuracy, a measure of how correct predicted pain levels based on machine learning are with actual NRS, using a confusion matrix (Table 3). As shown in Table 3, each row of the matrix represents the actual quartile NRS levels reported by patients, while each column represents predicted quartile pain levels based on machine learning. Using this matrix, we calculated Accuracy using identical sets of “levels” in both dimensions to be 79.6% (Table 4). Changes in pain levels before and after THA surgery as determined by NRS and EEG were positively correlated (Table 5).

**Table 5:** Pain levels were determined by NRS at pre and posy surgery, and subdivided into truth None, Mild, Moderate and Sever. Pain levels were also predicted by machine learning, and subdivided into quartile, and were compared with those by NRS.

Sex	NRS Pre	NRS Post	Truth Pre	Truth Post	Prediction Pre	Prediction Post
F	10	0	Severe	None	Severe	None
F	5	0	Moderate	None	Moderate	None
F	5	0	Moderate	None	Moderate	None
F	4	4	Moderate	Moderate	Moderate	Moderate
F	5	0	Moderate	None	Moderate	None
F	7	0	Severe	None	Severe	None
F	5	0	Moderate	None	Moderate	None
F	9	0	Severe	None	Severe	None
F	5	0	Moderate	None	Moderate	None
M	6	1	Moderate	Mild	Moderate	Mild
F	6	0	Moderate	None	Moderate	None
M	2	2	Mild	Mild	Mild	**Moderate**
F	3	0	Mild	None	Mild	None
F	7	0	Severe	None	**Moderate**	None
F	1	0	Mild	None	Mild	None
M	10	0	Severe	None	Severe	None
F	5	2	Moderate	Mild	Moderate	Mild
F	9	0	Severe	None	Severe	None
M	5	3	Moderate	Mild	Moderate	**Moderate**
F	8	0	Severe	None	Severe	None
M	6	1	Moderate	Mild	Moderate	Mild
F	4	0	Moderate	None	Moderate	None
F	4	4	Moderate	Moderate	Moderate	Moderate

Bold indicate prediction discrepancies between NRS and EEG findings.

## Discussion

To date, various methods have been applied to evaluate individual pain objectively, among them functional MRI or devices using weak electric currents^19^; but evaluation of pain using these strategies is limited to individuals at rest. Pain is induced and often worsens with motion. Such pain limits individuals’ QOL and ADL, and thus, evaluation and classification of patients’ pain as they perform various tasks is mandatory to treat these patients. EEG is non-invasive and can be recorded during ADL, but is easily affected by artifacts such as motion artifacts and EMG^25,27^. Indeed, previous studies characterizing chronic pain by EEG were applied using at least four electrodes to monitor pain at rest or during cognitive tasks^21,22^, and the pain during walking was not analyzed. Here, subjects’ brain waves were recorded using a single electrode in a wearable device, and data was electronically transferred to an iPAD, allowing subjects to walk freely as we recorded EEG during movement that resembled ADL. Moreover, a bandpass filter was adopted such that EMG was eliminated from EEG raw data, allowing each individual EEG feature to be analyzed. Furthermore, pain levels were evaluated by analyzing EEG features by machine learning with 79.6% Accuracy. In general, > 60% is considered high enough for Precision, Recall and Accuracy, and our results satisfied these criteria. Taken together, our system enables us to evaluate individual hip pain by monitoring EEG. This system could be useful to characterize patients’ pain, such as low back pain and OA, during ADL, to determine whether operative therapy is indicated, or to monitor effects of pain treatment.

Limitations in ADL due to pain are a serious health problem. However, in OA patients, there is frequently a discrepancy between an individual’s report of pain and radiographic findings^32,33,35^. Some patients feel pain with catastrophic thinking, and such pain is often resistant to operative therapy^42–44^. Several pain-catastrophizing scales based on questionnaires have been developed to characterize a patient’s pain and are reportedly useful to determine an indication for operative therapy^43,44^. Combining radiography and catastrophizing scales with our system could provide the best means to characterize a patient’s pain.

**Figure 5:**
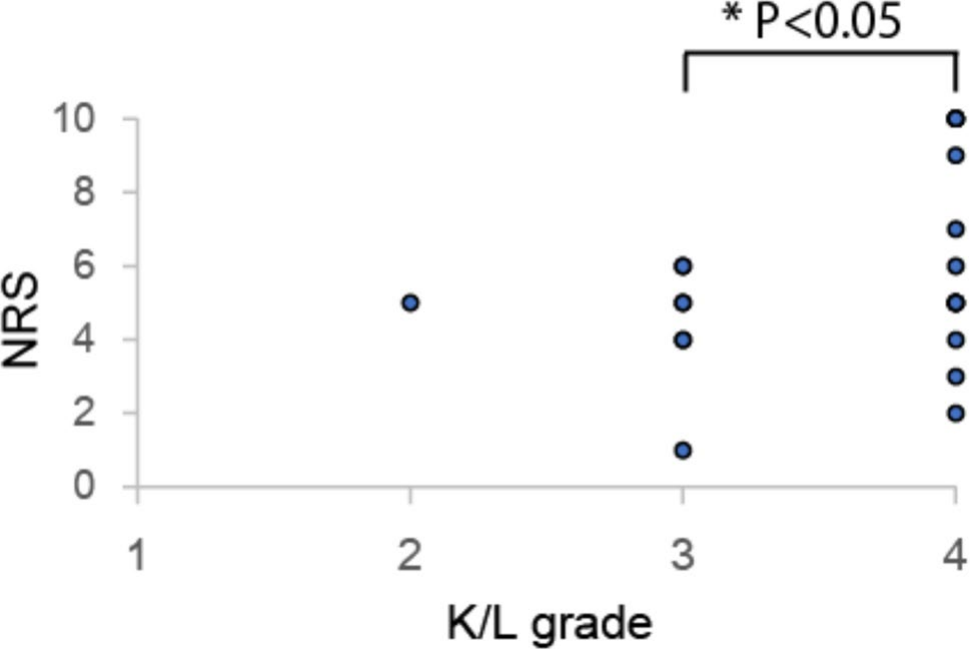
Kellgren/Lawrence grades are associated with NRS before THA surgery. Hip pain, as determined by NRS, was significantly greater in patients with K/L = 4 than those with K/L = 3. One osteonecrosis patient was excluded from analysis because their K/L grade was a classification adapted to OA, and osteonecrosis is known to cause strong pain with less radiographic change.

Our study has some limitations. Our subject number was small and limited to patients with hip pain. Postoperative results are reportedly more favorable in patients with THA than with total knee arthroplasty^45–47^, and thus we limited the study to subjects who underwent THA surgery. Ours was the first study to use this system, and it was necessary to limit the study to patients with apparent pain that could be relieved by therapy to assess EEG in subjects with or without pain. Moreover, various factors serve as an indication for THA surgery, among them radiographic findings such as a KL classification ≥ 3, and limited ADL of patients not caused by pain. In our subjects, except for osteonecrosis patients, OA grades determined by KL classification were ≥ 3, and others have reported that KL classification is significantly associated with hip pain^39–48^. Indeed, among our subjects, KL classification in OA patients was positively correlated with hip pain (Fig. 5). Moreover, ADL limitations are reportedly strongly associated with hip pain^39,48–50^. Thus, patients with hip pain owing to OA and who had undergone THA surgery were considered to meet these criteria. Finally, the ratio of females to males was relatively high in our study. In general, OA and osteonecrosis in hip joints are more common in females than males^38–40^, and indeed, more female patients underwent THA surgery at our hospital than males from 2017 to 2019. Thus, the ratio of females/males assessed here reflects the overall gender ratio of these diseases. Nonetheless, when we subdivided subjects into female and male groups and analyzed Accuracy, overall classification accuracies were higher in male than female groups (Supplementary Table 2–5, female 84.2%, male 95.0%), but both exceeded 60%. Furthermore, when we newly enrolled male subjects, we found that overall classification accuracy as determined by machine learning was 68.3% (Supplementary Table 6 and 7). Thus, we consider our method equally applicable to both genders.

Overall, we conclude that our system is a useful tool to monitor patients’ pain during ADL non-invasively and objectively.

## Supplementary Information

Supplementary Information.
