# Supplementary material for: Objective characterization of hip pain levels during walking by combining quantitative electroencephalography with machine learning
Source: Sci Rep. 2021 Feb 4;11:3192. doi: 10.1038/s41598-021-82696-1 (PMC7862297; doi:10.1038/s41598-021-82696-1)
Supplement: Supplementary file 1 — Supplementary Information. [file 41598_2021_82696_MOESM1_ESM.pdf]

Objective characterization of hip pain levels during walking by combining quantitative electroencephalography with machine learning

Atsushi Kimura[1], Yasue Mitsukura[4], Akihito Oya[1], Morio Matsumoto[1], Masaya Nakamura[1], Arihiko Kanaji[1] and Takeshi Miyamoto[1, 2,3, 5*]

[1]Department of Orthopedic Surgery, [2]Department of Advanced Therapy for Musculoskeletal Disorders II, [3]Department of Musculoskeletal Reconstruction and Regeneration Surgery, Keio University School of Medicine, 35 Shinano-machi, Shinjuku-ku, Tokyo 160-8582, Japan, [4] Department of Technology and Engineering, Keio University, Yokohama 2238532, Japan, [5]Department of Orthopedic Surgery, Kumamoto University, 1-1-1 Honjo, Chuo-ku, Kumamoto 860-8556, Japan

Correspondence should be addressed to T. M., or A.K., Department of Orthopedic Surgery, Keio University School of Medicine, 35 Shinano-machi, Shinjuku-ku, Tokyo 160-8582, Japan

TEL: 81-3-5363-3812, FAX: 81-3-3353-6597, e-mail: Takeshi Miyamoto (miyamoto@z5.keio.jp or miyamoto.takeshi@kuh.kumamoto-u.ac.jp)

Supplementary Table1

Parameters	N = 40
Age at THA(y)	64.5±12.3 (25-81)
Female/Male, n(%)	33(82.5)/7(17.5)
Height(cm)	156±7.45(144-172)
Body weight(kg)	55.0±7.45(36.7-73.2)
BMI(kg/m2)	22.7±3.7 (15.4-31.0)
Severity of OA(K/L grade).1/2/3/4,n(%)	1(2.5)/2(5.0)/15(37.5)/22(55.0)
OA due to DDH, n(%)	18(45.0)
Contralateral hip, healthy/OA/after THA, n(%)	13(32.5)/15(37.5)/12(30.0)

Characteristics of patients. Continuous values are expressed as mean ± standard deviation (range).
THA, total hip arthroplasty; BMI, body mass index; OA, osteoarthritis; K/L, kellgren-Lawrence; DDH, developmental dysplasia of the hip.

Supplementary Table2

		Output results			
		**None**	**Mild**	**Moderate**	**Severe**
Ground truth label	None	133	1	12	4
	Mild	3	24	3	0
	Moderate	14	2	101	3
	Severe	14	1	0	45

Results of pain levels classification using the SVM classifier (Female only). Each results were determined by dividing the number of identical sets of predicted (by the machine learning) and actual (NRS) pain levels by whole number of sets in each predicted or actual pain level, respectively.

Supplementary Table3

	**Precision**	**Recall**	**F1-score**	**Overall CA**
None	0.8110	0.8867	0.8471	0.842
Mild	0.8571	0.8000	0.8276	
Moderate	0.8707	0.8417	0.8559	
Severe	0.8654	0.7500	0.8036	

Values of the generalization performance of the classification using the SVM classifier (Female only). CA: classification accuracy.

Supplementary Table4

		**Output results**			
		**None**	**Mild**	**Moderate**	**Severe**
Ground truth label	None	9	0	1	0
	Mild	0	48	2	0
	Moderate	0	2	28	0
	Severe	0	0	0	10

Results of pain levels classification using the SVM classifier (Male only). Each results were determined by dividing the number of identical sets of predicted (by the machine learning) and actual (NRS) pain levels by whole number of sets in each predicted or actual pain level, respectively.

Supplementary Table5

	**Precision**	**Recall**	**F1-score**	**Overall CA**
None	1.0000	0.9000	0.9474	0.950
Mild	0.9600	0.9600	0.9600	
Moderate	0.9032	0.9333	0.9180	
Severe	1.0000	1.0000	1.0000	

Values of the generalization performance of the classification using the SVM classifier (Male only). CA: classification accuracy.

Supplementary Table6

		Output results			
		**None**	**Mild**	**Moderate**	**Severe**
Ground truth label	None	13	0	6	1
	Mild	0	0	0	0
	Moderate	5	0	24	1
	Severe	1	0	5	4

Results of pain levels classification using the SVM classifier (extra subjects). Each results were determined by dividing the number of identical sets of predicted (by the machine learning) and actual (NRS) pain levels by whole number of sets in each predicted or actual pain level, respectively.

Supplementary Table7

	Precision	Recall	F1-score	Overall CA
None	0.6500	0.6842	0.6667	0.6833
Mild	-	-	-	
Moderate	0.8000	0.6857	0.7385	
Severe	0.4000	0.6667	0.5000	

Values of the generalization performance of the classification using the SVM classifier (extra subjects). CA: classification accuracy.
